# Evaluation of Influenza Vaccine Effectiveness from 2021 to 2024: A Guangdong-Based Test-Negative Case–Control Study

**DOI:** 10.3390/vaccines13010004

**Published:** 2024-12-24

**Authors:** Liyan Zhu, Ying Han, Jiahai Lu, Jianhao Tan, Conghui Liao, Cheng Guo, Qing He, Yajie Qiu, Huahua Lu, Yue Zhou, Jianrui Wei, Dandan Hu

**Affiliations:** 1 Department of Child Healthcare, Guangzhou Women and Children’s Medical Center, Guangzhou Medical University, Guangzhou 510623, China; 2022210202@stu.gzhmu.edu.cn (L.Z.); hanying@gwcmc.org (Y.H.); tanjianhao@gwcmc.org (J.T.); heqing@gwcmc.org (Q.H.); qiuyajie@gwcmc.org (Y.Q.); 2023211124@stu.gzhmu.edu.cn (H.L.); 2024211154@stu.gzhmu.edu.cn (Y.Z.); 2 School of Public Health, Sun Yat-Sen University, Guangzhou 510080, China; lujiahai@mail.sysu.edu.cn (J.L.); lliaoch3@mail2.sysu.edu.cn (C.L.); guoch36@mail.sysu.edu.cn (C.G.); 3 National Medical Products Administration Key Laboratory for Quality Monitoring and Evaluation of Vaccines and Biological Products, Guangzhou 510080, China; 4 One Health Center of Excellence for Research & Training, Sun Yat-Sen University, Guangzhou 510080, China; 5 School of Laboratory Medicine and Life Science, Wenzhou Medical University, Wenzhou 325000, China; 6 Hainan Key Novel Thinktank “Hainan Medical University ‘One Health’ Research Center”, Haikou 571199, China; 7 Research Institute of Sun Yat-Sen University in Shenzhen, Shenzhen 518057, China; 8 Key Laboratory of Tropical Diseases Control, Sun Yat-Sen University, Ministry of Education, Guangzhou 510080, China; 9 Institute of One Health, Wenzhou Medical University, Wenzhou 325000, China; 10 Guangzhou Key Laboratory of Child Neurodevelopment, Guangzhou Women and Children’s Medical Center, Guangzhou Medical University, Guangzhou 510623, China

**Keywords:** influenza, vaccination, test-negative, case–control, China

## Abstract

Background: The influenza virus’s high mutation rate requires the annual reformulation and administration of the vaccine. Therefore, its vaccine effectiveness (VE) must be evaluated annually. Aim: Estimate the effectiveness of the influenza vaccine and analyze the impact of age, seasonal variations, and the vaccination to sample collection interval on VE. Methods: The study used a test-negative case–control (TNCC) design to collect data from patients under 18 years of age who presented with acute respiratory infection (ARI) symptoms and underwent influenza virus testing at a national children’s regional medical center in Guangdong Province between October 2021 and January 2024, spanning three influenza seasons. VE was estimated using unconditional logistic regression. Results: A total of 27,670 patient data entries were analyzed. The VE against all influenza viruses across the three seasons was 37% (95% CI: 31–43), with the lowest VE of 24% (95% CI: 8–37) observed in the 2021–2022 season. In children aged 0.5 to <3 years, the VE was 32% (95% CI: 19–43). The effectiveness for samples collected at intervals of 0.5–2 months, 3–6 months, and over 6 months after vaccination was 39% (95% CI: 32–46), 30% (95% CI: 19–40), and 28% (95% CI: 5–46). Conclusions: Across three influenza seasons, at least one-third of vaccinated individuals were protected from influenza in outpatient settings. Given that children are at high risk, improving vaccination management is recommended, and parents should be encouraged to vaccinate their children before each influenza season.

## 1. Introduction

The influenza virus, with its genetic variability and host diversity, causes epidemics marked by seasonal, regional, and subtype variations. These traits burden public health systems and may influence global pandemic patterns [1]. The primary approach to mitigating influenza and decreasing the incidence of severe illness and mortality associated with the virus is vaccination [2]. As a high-risk group for influenza, children particularly benefit from vaccination. In addition to providing direct protection against infection, vaccination also offers indirect protection to the wider community through herd immunity [3,4]. In mainland China, influenza vaccines are voluntary and self-paid, falling outside of the national immunization program. Given the regional differences in the timing and duration of influenza activity peaks each year, hospitals and clinics providing vaccination services are required to offer the influenza vaccine throughout the entire flu season. This ensures that recipients can receive immunity before the onset of peak influenza activity [5]. The influenza vaccines used in China include trivalent inactivated vaccines (TIVs), quadrivalent inactivated vaccines (QIVs), and trivalent live attenuated vaccines (LAIVs), all of which incorporate Northern Hemisphere strains to target the circulating influenza viruses.

The influenza virus, a negative-sense RNA virus, is distinguished by its complex classification, multiple subtypes, and high mutation rate. To ensure sustained vaccine efficacy, the World Health Organization (WHO) updates the influenza vaccine strain composition annually. As a result, vaccine effectiveness (VE) must be assessed each year to provide real-world evidence that is pivotal in informing public health decision-making [6]. In recent years, the global outbreak of COVID-19 and the subsequent epidemic control measures have significantly impacted influenza activity. Since March 2020, influenza levels in China remained extremely low until the onset of the 2021–2022 season, when both southern and northern provinces began to experience an increase in influenza cases [7].

This study aims to evaluate the real-world effectiveness of influenza vaccines during the three influenza seasons from 2021 to 2024, focusing on the influence of age differences, seasonal variations, vaccination timing, and vaccination completeness on VE. Using a case–control study, we examined the influence of these factors on VE, with particular emphasis on the importance of annual immunization and complete vaccination, especially for children as a high-risk group. The findings will provide scientific evidence to optimize vaccination management strategies, increase vaccination coverage rates, refine policies, and enhance public health outcomes.

## 2. Materials and Methods

### 2.1. Study Design and Study Population

Guangzhou Women and Children’s Medical Center serves as the National Regional Medical Center for Children. Patients are drawn from the Central South area of China, which ensures that the data are representative of this region.

This study collected data from October 2021 to January 2024, covering three influenza seasons (2021–2022, 2022–2023, and 2023–2024) for all patients under 18 years of age who sought influenza virus testing at outpatient clinics due to symptoms of acute respiratory infection (ARI).

The study utilized a test-negative case–control (TNCC) design, using influenza virus positive cases as the case group and negative cases as the control group. Included patients had to have ARI symptoms clearly labeled by their physician on the laboratory requisition form, and patients had to have a throat swab specimen collected at the hospital, which was later tested for influenza virus by real-time reverse transcription polymerase chain reaction (rRT-PCR) at the hospital’s central laboratory. If the same patient underwent two or more influenza virus tests within 14 days, only the first result was retained.

### 2.2. Sources of Information

The patient’s gender, date of birth, disease diagnosis, date of sample collection, influenza virus test results, and vaccination history, including vaccine type and timing, were collected from the electronic medical record system. Complete vaccination was defined as follows: (1) A patient under 9 years of age who had received 2 doses of inactivated vaccine that season, or was receiving 1 dose but had previously received 2 doses in prior seasons, or had received 1 dose of LAIV; and (2) a patient aged 9 years or older who had received 1 dose of influenza vaccine in the current season. Children requiring two doses but receiving only one were considered partially vaccinated. Cases without vaccination records or those with an age under 0.5 years at the first dose were excluded. Additionally, as studies have shown that protective antibodies typically develop 2–4 weeks after influenza vaccination [5], cases vaccinated less than 14 days prior to testing were also excluded. This exclusion criterion ensures sufficient time for immune responses to develop, prevents the potential underestimation of VE, and enhances the accuracy and validity of the results.

### 2.3. Statistical Analysis

Vaccine effectiveness (VE) can be calculated using the formula below:

VE = (1 − OR) × 100%

The odds ratio (OR) represents the ratio of the probability of vaccination between the case and control groups. Considering age-related differences in immune responses in children and the seasonal transmission of the influenza virus, factors such as age and the month of sample collection may confound the influenza vaccine’s protective effect [8]. To control for these confounders and improve result accuracy, we used an unconditional logistic regression model to estimate VE, adjusting for covariates such as sex, age, and the calendar month of sample collection. We calculated VE and its 95% confidence interval (95% CI) based on the OR value using the formula. VE was regarded as statistically significant when the 95% CIs did not overlap with zero. Although the confounding effect of sex on VE remains unclear, many studies [9,10] include sex as a covariate; thus, we retained sex as a potential confounder to ensure no influential factors were omitted.

VE was estimated separately for different influenza virus subtypes (Type A and Type B), across influenza seasons (2021–2022, 2022–2023, and 2023–2024), and for various age groups (0.5 to <3 years, 3 to <9 years, and 9 to <18 years). VE was estimated for the current and the previous three influenza seasons, with categories including: never vaccinated, vaccinated only in the current season, vaccinated in prior seasons but not in the current season, and vaccinated in both the current and prior seasons. VE was assessed based on the interval between vaccination and sample collection (0.5 to 2 months, 3 to 6 months, and over 6 months), as well as based on the status of complete and partial vaccination. Additionally, VE was evaluated for different vaccine types administered, including LAIV, TIV, and QIV.

We used the chi-square test to assess the differences in demographic, clinical diagnosis, and vaccination characteristics between the positive case and negative control group. Continuous variables were reported as medians with interquartile ranges (IQR), and categorical variables as frequencies and percentages. Statistical tests, conducted as two-tailed, were considered significant for *p*-values < 0.05. R software version 4.3.3 was used for all analyses.

## 3. Results

### 3.1. Participant Profiles

During the three influenza seasons, 27,670 patients with ARI received testing and 11,128 (40.2%) were diagnosed with influenza, of which 7839 (70.4%) had influenza A and 3976 (35.7%) had influenza B. During the 2023–2024 season, the test-positive group had a higher proportion of cases than the test-negative control group (66.3% vs. 55.4%, *p* < 0.001). the proportion of cases under 3 years of age was lower in the test-positive group compared to the control group (11.8% vs. 20%, *p* < 0.001). No significant differences were found between the test-positive and test-negative groups regarding gender. Moreover, a higher proportion of upper respiratory tract infections was observed in the test-positive group compared to the test-negative group (71.7% vs. 64.9%, *p* < 0.001). From December to January, the proportion of cases in the test-positive group was higher than the test-negative group (50.6% vs. 39.8%, *p* < 0.001). Among patients vaccinated against influenza in the current season, the test-positive group had a lower proportion than the test-negative group (7.6% vs. 12.2%, *p* < 0.001). Furthermore, regarding vaccination history in the current and prior seasons, a higher proportion of patients in the test-positive group were unvaccinated than the control group (67.5% vs. 62.1%, *p* < 0.001) (Table 1).

### 3.2. Profiles of Vaccine Recipients in the Current Season

Among the patients who received the influenza vaccine in each flu season (10.3%), the proportion of the test-positive group with an interval of 3 to 6 months between vaccination and sample collection was greater as compared to the control group (34.2% vs. 32.0%, *p* < 0.001). Furthermore, the proportion of individuals who were completely vaccinated in the test-positive group was lower than in the control group (49.8% vs. 51.7%, *p* < 0.001). In contrast, a higher proportion of individuals in the test-positive group received the QIV compared to the control group (78.8% vs. 73.3%, *p* = 0.002) (Table 2).

### 3.3. Influenza Vaccine Effectiveness by Age Group

**Figure 1:**
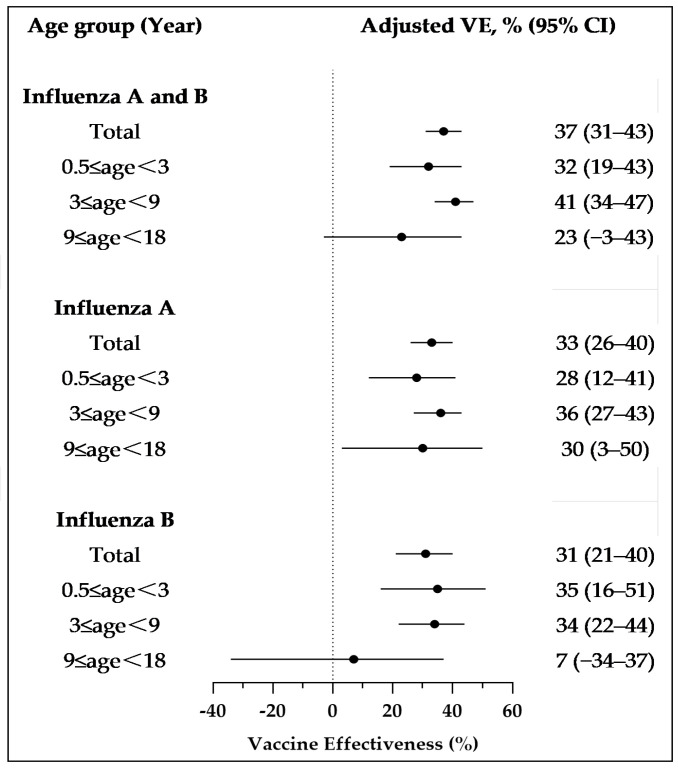
Vaccine effectiveness against outpatient cases from October 2021 to January 2024, across different influenza types and age groups. Results were adjusted for gender, age group, and sample collection month. Abbreviations: CI, confidence interval; VE, vaccine effectiveness.

In Guangzhou, influenza activity occurs almost year-round. Considering only the current season’s vaccination, the overall VE across all influenza types over three years was 37% (95% CI: 31–43). The age group of 3 to < 9 years exhibited the highest VE at 41% (95% CI: 34–47). The VE for the 0.5 to < 3 years age group was 32% (95% CI: 19–43), whereas the VE for the 9 to < 18 years age group was not statistically significant, at 23% (95% CI: −3 to 43). For influenza A, the overall VE was 33% (95% CI: 26–40), with the VE in the three age groups (0.5 to <3 years, 3 to <9 years, and 9 to <18 years) being 28% (95% CI: 12–41), 36% (95% CI: 27–43), and 30% (95% CI: 3–50). For influenza B, the overall VE was 31% (95% CI: 21–40), with age-specific VEs of 35% (95% CI: 16–51), 34% (95% CI: 22–44), and 7% (95% CI: −34 to 37) (Figure 1).

### 3.4. Vaccine Effectiveness Across Influenza Seasons

**Figure 2:**
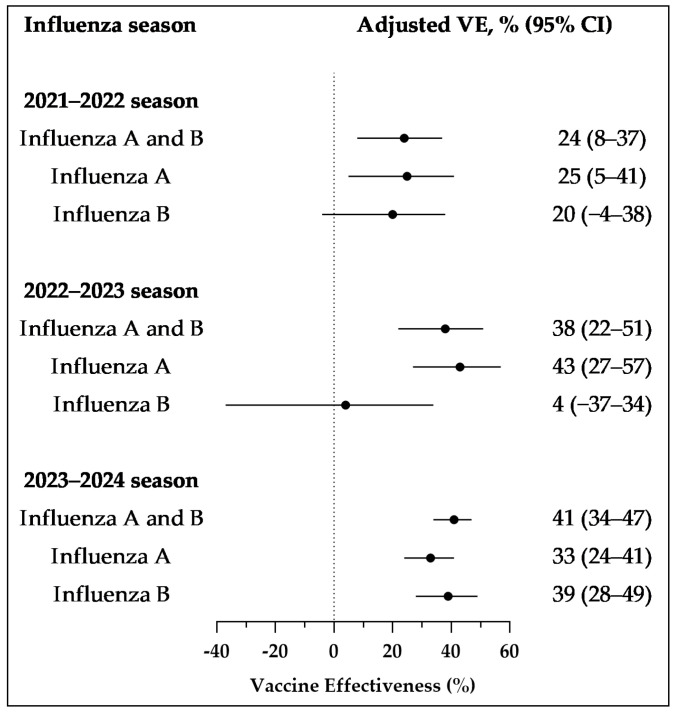
Vaccine effectiveness against outpatient cases from October 2021 to January 2024, across different influenza seasons and types. Results were adjusted for gender, age group, and sample collection month. Abbreviations: CI, confidence interval; VE, vaccine effectiveness.

For all influenza types, the VE was lowest during the 2021–2022 season at 24% (95% CI: 8–37), while the VE for the 2022–2023 and 2023–2024 periods was 38% (95% CI: 21–51) and 41% (95% CI: 34–47). For influenza A, the VE for each season (2021–2022, 2022–2023, and 2023–2024) was 25% (95% CI: 5–41), 43% (95% CI: 27–57), and 33% (95% CI: 24–41). For influenza B, the VEs for the three influenza seasons were 20% (95% CI: −4 to 38), 4% (95% CI: −37 to 34), and 39% (95% CI: 28–49) (Figure 2).

### 3.5. Vaccine Effectiveness Estimates Under Different Vaccination Scenarios

**Figure 3:**
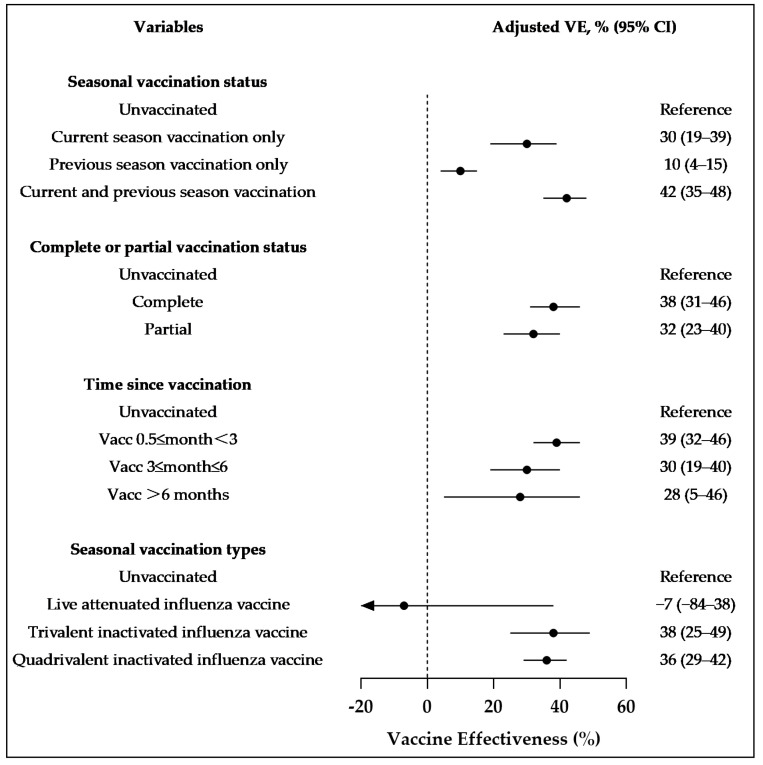
Vaccine effectiveness against outpatient cases of all influenza types from October 2021 to January 2024, across different influenza vaccination characteristics. Results were adjusted for gender, age group, and sample collection month. Abbreviations: CI, confidence interval; VE, vaccine effectiveness.

Compared to individuals who had not received the vaccine in the current and prior seasons, the VE for those vaccinated only in the present season was 30% (95% CI: 19–39). In contrast, VE for those vaccinated in prior seasons was only 10% (95% CI: 4–15). The highest VE, 42% (95% CI: 35–48), was found in participants vaccinated in both the present and prior seasons. Compared to those who were not vaccinated in the current season, VE decreased with an increasing interval between vaccination and sample collection. The VEs for intervals of 0.5 to 2 months, 3 to 6 months, and over 6 months were 39% (95% CI: 32–46), 30% (95% CI: 19–40), and 28% (95% CI: 5–46). Individuals with complete vaccination had a higher VE compared to those who were partially vaccinated, with values of 38% (95% CI: 31–46) and 32% (95% CI: 23–40). The VEs for TIV and QIV administered in the current season were similar, at 38% (95% CI: 25–49) and 36% (95% CI: 29–42) (Figure 3).

## 4. Discussion

Our study demonstrated that over the course of three seasons, the overall VE against all influenza types, as well as against influenza A and B, was 37%, 33%, and 31%, respectively. These results indicate that influenza vaccination provides moderate protection.

### 4.1. Strengthening Vaccination Management for High-Risk Populations

The study demonstrated that influenza vaccines provided lower protective effectiveness in children under the age of three compared to those aged three years or older, aligning with the observations reported by Fu [11] and Feng [12]. This highlights the importance of prioritizing vaccination efforts for young children. Studies show that children under 5 years old, particularly those under 2 years old, are at a higher risk of hospitalization, serious complications, and mortality after contracting the influenza virus [13,14,15,16]. Studies by Alshehri [17] and Atikel [18] have demonstrated that individuals at higher risk for severe flu or complications are more inclined to skip influenza vaccination. Therefore, we recommend enhancing influenza vaccination management for populations under 5 years old, especially those under 2 years old. The Technical Guidelines for Influenza Vaccination in China (2023–2024) also suggest prioritizing influenza vaccination for children aged 0.5 to 5 years [5]. The U.S. Advisory Committee on Immunization Practices (ACIP) also designates this group as a top priority for seasonal flu vaccination [19]. To improve vaccination management among high-risk groups, it is crucial to conduct public education campaigns to raise awareness about the importance of the influenza vaccine. Furthermore, training for healthcare providers should be strengthened to enhance their motivation and effectiveness in recommending vaccines, while also emphasizing the necessity of vaccination in medical documentation to encourage patients to receive the vaccine during routine visits.

### 4.2. Vaccine Strain Mismatch Affects VE

Influenza VE exhibits seasonal variation, with protective effectiveness against all influenza viruses recorded at 24%, 38%, and 41% for the 2021–2022, 2022–2023, and 2023–2024 influenza seasons.

The overall VE for the 2021–2022 season was relatively low in the Northern Hemisphere, consistent with studies from Denmark, Europe, the United States, and Spain [20,21,22,23]. This may be due to a mismatch between the vaccine strain, based on predicted virus strains, and the circulating strains. A study conducted in Spain during the 2021–2022 season found that the predominant influenza A(H3N2) strain belonged to the 3C.2a1b.2a.2 sublineage, which exhibited antigenic differences compared to the 3C.2a1b.2a.1 strain used in the vaccine [23]. Additionally, reports from the China Influenza Center showed that 77.2% of A(H3N2) influenza virus strains in the 2021–2022 season were similar to the A/Darwin/9/2021 (egg-based vaccine) strain, further confirming the issue of vaccine component mismatch [24].

The vaccine strains in other seasons were generally well-matched to the circulating strains. According to the Chinese Influenza Weekly Report, during the 2022–2023 flu season, 98.2% of the dominant influenza A(H1N1)pdm09 strains were similar to the A/Victoria/2570/2019 vaccine strain. In the 2023–2024 flu season, 99.1% of the circulating B-type influenza strains resembled the B/Austria/1359417/2021 vaccine strain. Nevertheless, there are variations in VE across different countries in the Northern Hemisphere [24]. During the 2022–2023 season, the VE in Europe and the United States was higher than that observed in our study, while the VE in Spain was lower [25,26,27]. In the 2023–2024 season, the VE in the United States and Italy was higher than that observed in our study, whereas the VE in South Korea was lower [28,29,30].

The influenza virus exhibits significant variability, with two key surface glycoproteins, hemagglutinin (HA) and neuraminidase (NA), playing crucial roles as immunogens that trigger protective antibody responses [31]. These proteins, as the main antigens of the virus, undergo continuous mutations through antigenic drift, which helps the virus escape recognition by the immune system. The process of antigenic drift results in alterations to the viral antigens, leading to mismatches between the vaccine strain and the strains circulating in the population. This lack of alignment hinders the immune system’s ability to recognize and neutralize the virus, thus diminishing the effectiveness of the vaccine [32]. This problem is particularly evident in the case of H3N2 influenza viruses, where the high mutation rate calls for regular updates to the vaccine strains. Between 2011 and 2021, vaccines were updated every year except for two periods: 2013–2015 and 2016–2018. The challenge of addressing the virus’s high mutation rate and selecting the most suitable vaccine strains persists. To improve the efficacy of influenza vaccines, strategies aimed at enhancing their broad-spectrum protective effects will be vital.

### 4.3. Vaccination Before Each Influenza Season

Given the varying circulating viruses and vaccine strains each influenza season, as well as the differences in VE, we recommend annual influenza vaccination. Our study also found that individuals vaccinated in both the previous and current seasons experienced the highest protective effect, reaching 42%. Furthermore, the vaccine from the previous season still offered a 10% protective effect during the current season. Similar findings have been reported in studies from Spain and Canada, further supporting the evidence for annual influenza vaccination efforts [23,33].

This study found that VE declines over time within the current season, decreasing from 39% within 2 months post-vaccination to 28% after 6 months. A study from Hong Kong covering the 2012–2013 to 2016–2017 seasons reported that VE in individuals under 18 years dropped from 79% within 2 months post-vaccination to 45% after 6–9 months [12]. A European study during the 2021–2022 season showed that for all age groups against influenza A (H3N2), VE declined from 54% within 3 months of vaccination to 16% after 5 months [21]. Because the effectiveness of vaccination decreases over time, we suggest receiving the vaccine before local influenza activity begins to ensure early immune protection. This recommendation aligns with the guidelines outlined in China’s influenza vaccination technical guidelines (2023–2024) [5].

Our study shows that the VE of the TIV and QIV in the current season is similar, at 38% and 36%, respectively. This indicates that administering the trivalent vaccine is sufficient to meet vaccination needs. The quadrivalent vaccine includes an additional B strain, specifically the Yamagata lineage. However, since March 2020, the global influenza surveillance network has not detected any Yamagata lineage strains. The World Health Organization has also repeatedly advised that current influenza vaccines should not include components from the Yamagata lineage [34]. Both trivalent and quadrivalent influenza vaccines provide the same level of protection, as long as the vaccine is administered.

### 4.4. Ensuring Complete Vaccination Coverage

Our study also found that, compared to those unvaccinated during the season, the VE for individuals who were fully vaccinated was higher at 38% compared to 32% for those who were partially vaccinated. Similar studies suggest that two doses at the start of the beginning influenza season provide optimal immunity [11,35]. Therefore, we recommend enhancing the coverage of the second dose of the influenza vaccine for children aged 6 months to 8 years who have not completed their vaccination schedule. Clinical practice should enhance follow-up efforts, providing timely reminders via phone or text messages to parents of children who have not received their second dose, and assisting in scheduling vaccination appointments until full vaccination is achieved.

### 4.5. Increasing Vaccination Coverage

Our study shows an influenza vaccine coverage of only 10.3%, much lower than in developed countries. In the UK, the coverage rate for all school-age children from 2021 to 2023 was approximately 50% [36], while the coverage for children under 18 in the US over the past three seasons is around 53.9% to 56.5% [37]. This coverage rate is even lower than the rates in China prior to the COVID-19 pandemic. A meta-analysis on influenza vaccine coverage in mainland China, primarily focusing on eastern urban areas, reported a vaccination rate of 25.1% among individuals under 18 years old from 2006 to 2016 [38]. Ensuring a high vaccination coverage rate is crucial for maintaining public health. When vaccination rates are insufficient, the likelihood of vaccine-preventable infectious diseases spreading in the broader community increases [39,40,41]. Additionally, outbreaks of these diseases can consume public health resources [42,43].

To increase influenza vaccine coverage, particularly in regions with low vaccination rates, such as China, effective public health strategies should be adopted, drawing on the experiences of countries like the UK and the US. First, it is recommended to include the influenza vaccine in the National Immunization Program, ensuring its widespread availability through policy support, especially for high-risk populations, such as the elderly, pregnant women, children, and individuals with chronic diseases, thereby reducing flu-related complications and mortality. Second, health education should be strengthened in communities, schools, and healthcare settings to reduce vaccine hesitancy, increase public awareness of the influenza vaccine, and eliminate misconceptions and biases. Additionally, temporary vaccination sites at the grassroots level should be established prior to the flu season, with extended service hours, or centralized vaccination events organized by employers to improve convenience, particularly for working adults and students. These measures will help reduce vaccination barriers, ensure the timely vaccination of at-risk groups, and further improve vaccine coverage.

### 4.6. Advantages and Limitations

One major strength of this study is the substantial sample size, which allows for the analysis of individual seasons, thereby enhancing the precision of the results and the statistical power. Additionally, it enables the investigation of VE over six months post-vaccination and includes data on vaccination status from previous seasons, contributing to a more comprehensive discussion.

However, there are several limitations in our study. Firstly, the lack of specific strain and genetic data on the influenza virus prevented us from assessing the potential variations in the match between vaccine strains and circulating strains. Future research should prioritize collecting and analyzing genetic information from influenza viruses, particularly to further investigate the influence of antigenic drift on VE. This would help optimize vaccine design, enhance influenza prevention and control strategies, and provide a more scientifically robust foundation for the development and adjustment of public health policies.

Secondly, the absence of data regarding COVID-19 infection status in our patients limited our ability to control for potential confounding biases introduced by COVID-19. Since both COVID-19 and influenza share similarities in transmission modes, clinical manifestations, and preventive measures, COVID-19 may affect the effectiveness and uptake of the influenza vaccine, especially when the two diseases co-circulate during the flu season. For example, COVID-19 control measures might reduce influenza transmission, thereby influencing VE. Moreover, co-infection with both COVID-19 and influenza could exacerbate immune system stress or provoke abnormal immune responses, further impacting the protective effect of the vaccine [44]. Consequently, future studies should explore how COVID-19 infection interferes with the efficacy of influenza vaccines, especially during periods of co-circulation, to provide evidence for optimizing vaccination strategies.

Furthermore, our VE analysis was limited to outpatient ARI cases, excluding severe influenza-related diseases. While outpatient data provide valuable insights into vaccine protection, analyzing hospitalized patients and influenza-related mortality would allow for a more comprehensive assessment of VE. Future research should expand the study population, particularly focusing on severe influenza cases, and incorporate data on complications and mortality to conduct a thorough TNCC analysis, thus enabling a more complete evaluation of the VE.

## 5. Conclusions

The study results show that approximately one-third of those vaccinated against influenza over the three seasons were protected from laboratory-confirmed influenza cases in outpatient settings. In the future, we will continue to investigate influenza vaccine effectiveness, providing evidence to support vaccination strategies, in order to better serve susceptible populations and enhance vaccine protection.

## Figures and Tables

**Table 1:** Demographics, clinical diagnosis, and vaccination characteristics of the study population [*n* (%)].

Variables	All Patients *n* (%) (*n* = 27,670)	Test-Positive Cases *n* (%) (*n* = 11,128)	Test-Negative Controls *n* (%) (*n* = 16,542)	*p*-Value ^1^
Influenza season				<0.001
2021–2022	6052 (21.9)	2081 (18.7)	3971 (24.0)	
2022–2023	5078 (18.4)	1669 (15.0)	3409 (20.6)	
2023–2024	16,540 (59.7)	7378 (66.3)	9162 (55.4)	
Age groups (years)				<0.001
0.5 ≤ age < 3	4628 (16.7)	1313 (11.8)	3315 (20)	
3 ≤ age < 9	16,884 (61)	6887 (61.9)	9997 (60.4)	
9 ≤ age < 18	6158 (22.3)	2928 (26.3)	3230 (19.6)	
Sex				0.628
Male	15,349 (55.5)	6193 (55.7)	9156 (55.4)	
Female	12,321 (44.5)	4935 (44.3)	7386 (44.6)	
Respiratory tract infection site				<0.001
Upper	18,712 (67.6)	7984(71.7)	10,728 (64.9)	
Lower	8958 (32.4)	3144(28.3)	5814 (35.1)	
Month of sample collection				<0.001
1	5130 (18.5)	2173 (19.5)	2957 (17.1)	
2	326 (1.2)	56 (0.5)	270 (1.7)	
3	3004 (10.9)	884 (7.9)	2120 (12.9)	
4	1958 (7.1)	753 (6.8)	1205 (7.4)	
5	578 (2.1)	188 (1.7)	390 (2.5)	
6	2039 (7.4)	1259 (11.3)	780 (4.8)	
7	604 (2.2)	206 (1.9)	398 (2.5)	
8	300 (1.1)	25 (0.2)	275 (1.8)	
9	562 (2.0)	85 (0.8)	477 (2.8)	
10	1254 (4.5)	248 (2.2)	1006 (6.1)	
11	4711 (17.0)	1792 (16.1)	2919 (17.7)	
12	7204 (26.0)	3459 (31.1)	3745 (22.7)	
Type of Influenza				
Influenza A	-	7833 (70.4)	-	
Influenza B	-	3976 (35.7)	-	
Vaccination status in the current season				<0.001
Unvaccinated	24,813 (89.7)	10,280 (92.4)	14,533 (87.8)	
Vaccinated	2857 (10.3)	848 (7.6)	2009 (12.2)	
Vaccination status in the current and previous seasons				<0.001
Unvaccinated	17,788 (64.3)	7511 (67.5)	10,277 (62.1)	
Current season only	1110 (4.0)	332 (3.0)	778 (4.7)	
Previous season only	7025 (25.4)	2769 (24.9)	4256 (25.7)	
Current and previous season	1747 (6.3)	516 (4.6)	1231 (7.5)	

^1^ We used the chi-square test to assess the differences in demographic, clinical diagnosis, and vaccination characteristics between the positive case group and negative control group.

**Table 2:** Vaccination characteristics of the population vaccinated in the current season [*n* (%)].

Variables	Vaccinated Patients *n* (%) (*n* = 2857)	Test-Positive Cases *n* (%) (*n* = 848)	Test-Negative Controls *n* (%) (*n* = 2009)	*p*-Value ^1^
Time since vaccination (months)				<0.001
Vacc 0.5 ≤ months < 3	1639 (57.4)	467 (55.1)	1172 (58.3)	
Vacc 3 ≤ months ≤ 6	933 (32.6)	290 (34.2)	643 (32.0)	
Vacc > 6 months	285 (10.0)	91 (10.7)	194 (9.7)	
Complete or partial vaccination				<0.001
Complete	1461 (51.1)	422 (49.8)	1039 (51.7)	
Partial	1396 (48.9)	426 (50.2)	970 (48.3)	
Seasonal vaccination types				<0.001
LAIV	57 (2.0)	24 (2.8)	33 (1.6)	
TIV	661 (23.1)	156 (18.4)	505 (25.1)	
QIV	2139 (74.9)	668 (78.8)	1471 (73.3)	

^1^ We used the chi-square test to assess the differences in vaccination characteristics between the positive case group and negative control group.

## Data Availability

The contributions of this study are included in the article. Any further inquiries should be directed to the corresponding author.

## References

[1] Kim Y.-H., Hong K.-J., Kim H., Nam J.-H. (2022). Influenza vaccines: Past, present, and future. Rev. Med. Virol..

[2] Buchy P., Badur S. (2020). Who and when to vaccinate against influenza. Int. J. Infect. Dis..

[3] Committee on Infectious Diseases (2023). Recommendations for Prevention and Control of Influenza in Children, 2023–2024. Pediatrics.

[4] Bonilla F.A., Khan D.A., Ballas Z.K., Chinen J., Frank M.M., Hsu J.T., Keller M., Kobrynski L.J., Komarow H.D., Mazer B. (2015). Practice parameter for the diagnosis and management of primary immunodeficiency. J. Allergy Clin. Immunol..

[5] TWG I.V., Committee N.I.A., Group T.W. (2023). Technical guidelines for seasonal influenza vaccination in China (2023–2024). Zhonghua Liuxingbingxue Zazhi.

[6] Tartof S.Y., Qian L., Liu I.-L.A., Tseng H.F., Sy L.S., Hechter R.C., Lewin B.J., Jacobsen S.J. (2019). Safety of influenza vaccination administered during hospitalization. Mayo Clinic Proc..

[7] Ilyicheva T., Kolosova N., Durymanov A., Torzhkova P.Y., Svyatchenko S., Bulanovich Y., Ivanova E., Ivanova K., Ryzhikov A. (2021). 2019–2020 herd immunity to seasonal influenza viruses prior to epidemic season and rate of severe disease cases. Russ. J. Infect. Immun..

[8] Chua H., Feng S., Lewnard J.A., Sullivan S.G., Blyth C.C., Lipsitch M., Cowling B.J. (2020). The Use of Test-negative Controls to Monitor Vaccine Effectiveness: A Systematic Review of Methodology. Epidemiology.

[9] Chard A.N., Nogareda F., Regan A.K., Barraza M.F.O., Fasce R.A., Vergara N., Avendaño M., Penayo E., Vázquez C., Von Horoch M. (2023). End-of-season influenza vaccine effectiveness during the Southern Hemisphere 2022 influenza season—Chile, Paraguay, and Uruguay. Int. J. Infect. Dis..

[10] Maurel M., Pozo F., Pérez-Gimeno G., Buda S., Sève N., Oroszi B., Hooiveld M., Gomez V., Domegan L., Martínez-Baz I. (2024). Influenza vaccine effectiveness in Europe: Results from the 2022–2023 VEBIS (Vaccine Effectiveness, Burden and Impact Studies) primary care multicentre study. Influenza Other Respir. Viruses.

[11] Fu C., Greene C.M., He Q., Liao Y., Wan Y., Shen J., Rong C., Zhou S. (2020). Dose effect of influenza vaccine on protection against laboratory-confirmed influenza illness among children aged 6 months to 8 years of age in southern China, 2013/14–2015/16 seasons: A matched case–control study. Hum. Vaccines Immunother..

[12] Feng S., Chiu S.S., Chan E.L.Y., Kwan M.Y.W., Wong J.S.C., Leung C.-W., Chung Lau Y., Sullivan S.G., Malik Peiris J.S., Cowling B.J. (2018). Effectiveness of influenza vaccination on influenza-associated hospitalisations over time among children in Hong Kong: A test-negative case-control study. Lancet Respir. Med..

[13] Shang M., Blanton L., Brammer L., Olsen S.J., Fry A.M. (2018). Influenza-associated pediatric deaths in the United States, 2010–2016. Pediatrics.

[14] Chaves S.S., Perez A., Farley M.M., Miller L., Schaffner W., Lindegren M.L., Sharangpani R., Meek J., Yousey-Hindes K., Thomas A. (2014). The burden of influenza hospitalizations in infants from 2003 to 2012, United States. Pediatr. Infect. Dis. J..

[15] Gill P.J., Ashdown H.F., Wang K., Heneghan C., Roberts N.W., Harnden A., Mallett S. (2015). Identification of children at risk of influenza-related complications in primary and ambulatory care: A systematic review and meta-analysis. Lancet Respir. Med..

[16] Hardelid P., Verfuerden M., McMenamin J., Gilbert R. (2017). Risk factors for admission to hospital with laboratory-confirmed influenza in young children: Birth cohort study. Eur. Respir. J..

[17] Alshehri A., Ahmed M., Bagazi D., Alghamdi A. (2023). Healthcare Providers’ Adherence to Recommended Pneumococcal and Influenza Vaccination in Patients Discharged with Respiratory Diseases from General Medical Wards. Vaccines.

[18] Atikel Y.Ö., EZGÜ S.A.B., Paglialonga F., Stefanidis C.J., Askiti V., Vidal E., Ariceta G., Melek E., Verrina E., Printza N. (2021). Influenza and pneumococcus vaccination rates in pediatric dialysis patients inEurope: Recommendations vs realityA European Pediatric Dialysis Working Group and European Society for PediatricNephrology Dialysis Working Group study. Turk. J. Med. Sci..

[19] Prevention and Control of Seasonal Influenza with Vaccines: Recommendations of the Advisory Committee on Immunization Practices–United States, 2024–2025 Influenza Season. https://www.cdc.gov/mmwr/volumes/72/rr/rr7202a1.htm?utm_campaign=&utm_medium=email&utm_source=govdelivery.

[20] Emborg H.-D., Vestergaard L.S., Botnen A.B., Nielsen J., Krause T.G., Trebbien R. (2022). A late sharp increase in influenza detections and low interim vaccine effectiveness against the circulating A (H3N2) strain, Denmark, 2021/22 influenza season up to 25 March 2022. Eurosurveillance.

[21] Kissling E., Pozo F., Martínez-Baz I., Buda S., Vilcu A.M., Domegan L., Mazagatos C., Dijkstra F., Latorre-Margalef N., Kurečić Filipović S. (2023). Influenza vaccine effectiveness against influenza A subtypes in Europe: Results from the 2021–2022 I-MOVE primary care multicentre study. Influenza Other Respir. Viruses.

[22] Price A.M., Flannery B., Talbot H.K., Grijalva C.G., Wernli K.J., Phillips C.H., Monto A.S., Martin E.T., Belongia E.A., McLean H.Q. (2023). Influenza vaccine effectiveness against influenza A (H3N2)-related illness in the United States during the 2021–2022 influenza season. Clin. Infect. Dis..

[23] Martínez-Baz I., Casado I., Miqueleiz A., Navascués A., Pozo F., Trobajo-Sanmartín C., Albéniz E., Elía F., Burgui C., Fernández-Huerta M. (2022). Effectiveness of influenza vaccination in preventing influenza in primary care, Navarre, Spain, 2021/22. Eurosurveillance.

[24] Summary of Influenza Prevalence in China Chinese National Infuenza Center. https://ivdc.chinacdc.cn/cnic/.

[25] Kissling E., Maurel M., Emborg H.-D., Whitaker H., McMenamin J., Howard J., Trebbien R., Watson C., Findlay B., Pozo F. (2023). Interim 2022/23 influenza vaccine effectiveness: Six European studies, October 2022 to January 2023. Eurosurveillance.

[26] McLean H.Q. (2023). Interim estimates of 2022–23 seasonal influenza vaccine effectiveness—Wisconsin, October 2022–February 2023. MMWR. Morb. Mortal. Wkly. Rep..

[27] Martínez-Baz I., Fernández-Huerta M., Navascués A., Pozo F., Trobajo-Sanmartín C., Casado I., Echeverria A., Ezpeleta C., Castilla J. (2023). Influenza vaccine effectiveness in preventing laboratory-confirmed influenza cases and hospitalizations in Navarre, Spain, 2022–2023. Vaccines.

[28] Zhu S. (2024). Interim Influenza Vaccine Effectiveness Against Laboratory-Confirmed Influenza—California, October 2023–January 2024. MMWR. Morb. Mortal. Wkly. Rep..

[29] Costantino C., Mazzucco W., Graziano G., Maida C.M., Vitale F., Tramuto F. (2024). Mid-Term Estimates of Influenza Vaccine Effectiveness against the A (H1N1) pdm09 Prevalent Circulating Subtype in the 2023/24 Season: Data from the Sicilian RespiVirNet Surveillance System. Vaccines.

[30] Choi Y.J., Sohn J.W., Choi W.S., Wie S.-H., Lee J., Lee J.-S., Jeong H.W., Eom J.S., Nham E., Seong H. (2024). Interim Estimates of 2023–2024 Seasonal Influenza Vaccine Effectiveness Among Adults in Korea. J. Korean Med. Sci..

[31] Wu N.C., Wilson I.A. (2020). Influenza Hemagglutinin Structures and Antibody Recognition. Cold Spring Harb. Perspect. Med..

[32] Wu N.C., Wilson I.A. (2017). A Perspective on the Structural and Functional Constraints for Immune Evasion: Insights from Influenza Virus. J. Mol. Biol..

[33] Nichols M., Andrew M., Ye L., Hatchette T., Ambrose A., Boivin G., Bowie W., Dos Santos G., Elsherif M., Green K. (2019). The impact of prior season vaccination on subsequent influenza vaccine effectiveness to prevent influenza-related hospitalizations over 4 influenza seasons in Canada. Clin. Infect. Dis..

[34] Recommended Composition of Influenza Virus Vaccines for Use in the 2023–2024 Northern Hemisphere Influenza Season. https://www.who.int/publications/m/item/recommended-composition-of-influenza-virus-vaccines-for-use-in-the-2023-2024-northern-hemisphere-influenza-season.

[35] Chung J.R., Flannery B., Gaglani M., Smith M.E., Reis E.C., Hickey R.W., Jackson M.L., Jackson L.A., Belongia E.A., McLean H.Q. (2020). Patterns of influenza vaccination and vaccine effectiveness among young US children who receive outpatient care for acute respiratory tract illness. JAMA Pediatr..

[36] UKHSA National Childhood Influenza Vaccination Programme 2022 to 2023. https://www.gov.uk/.

[37] Influenza Vaccination Coverage, Children 6 months through 17 years, United States. https://www.cdc.gov/fluvaxview/dashboard/children-vaccination-coverage.html.

[38] Wang Q., Yue N., Zheng M., Wang D., Duan C., Yu X., Zhang X., Bao C., Jin H. (2018). Influenza vaccination coverage of population and the factors influencing influenza vaccination in mainland China: A meta-analysis. Vaccine.

[39] Robison S.G., Liko J. (2017). The timing of pertussis cases in unvaccinated children in an outbreak year: Oregon 2012. J. Pediatr..

[40] Glanz J.M., McClure D.L., Magid D.J., Daley M.F., France E.K., Salmon D.A., Hambidge S.J. (2009). Parental refusal of pertussis vaccination is associated with an increased risk of pertussis infection in children. Pediatrics.

[41] Hesse E.M. (2019). Notes from the Field: Administration of expired injectable influenza vaccines reported to the vaccine adverse event reporting system—United States, July 2018–March 2019. MMWR. Morb. Mortal. Wkly. Rep..

[42] Rosen J.B., Arciuolo R.J., Khawja A.M., Fu J., Giancotti F.R., Zucker J.R. (2018). Public health consequences of a 2013 measles outbreak in New York City. JAMA Pediatr..

[43] Lina B., Georges A., Burtseva E., Nunes M.C., Andrew M.K., McNeil S.A., Ruiz-Palacios G.M., Feng L., Kyncl J., Vanhems P. (2020). Complicated hospitalization due to influenza: Results from the Global Hospital Influenza Network for the 2017–2018 season. BMC Infect. Dis..

[44] Su Y., Guo Z., Gu X., Sun S., Wang K., Xie S., Zhao S. (2023). Influenza vaccine effectiveness against influenza A during the delayed 2022/23 epidemic in Shihezi, China. Vaccine.

